# ﻿A new species related to *Pardosaatrata* (Araneae, Lycosidae) from Armenia makes the distribution range of the *atrata* group disjunctive

**DOI:** 10.3897/zookeys.1180.111069

**Published:** 2023-09-21

**Authors:** Yuri M. Marusik

**Affiliations:** 1 Institute for Biological Problems of the North FEB RAS, Portovaya Str. 18, Magadan 685000, Russia Institute for Biological Problems of the North FEB RAS Magadan Russia; 2 Altai State University, Lenina Pr., 61, Barnaul, RF-656049, Russia Altai State University Barnaul Russia; 3 Department of Zoology & Entomology, University of the Free State, Bloemfontein 9300, South Africa University of the Free State Bloemfontein South Africa

**Keywords:** Caucasus, Holarctic, Nearctic, new synonym, Palaearctic, Pardosini

## Abstract

Species of wolf spiders considered in the *Pardosaatrata* group are surveyed, and comparative figures of all species included in the group are presented for the first time. A new species, *P.armeniaca***sp. nov.**, is described from the shore of Lake Sevan (Armenia) based on both sexes. *Pardosanarymica* Savelyeva, 1972 from East Kazakhstan Oblast’ is synonymized with the trans-Palaearctic *P.atrata* (Thorell, 1873). It was found that the record of *P.atrata* (sub *P.lapponica*) by Schenkel from British Columbia and reflected in the World Spider Catalog in fact refers to *P.concinna* (Thorell, 1877), a member of the *P.lapponica* group. The distribution of three species consideredin the *atrata* group are mapped. The discovery of *P.armeniaca***sp. nov.** has led to a re-evaluation of the distribution range, previously thought to be continuous, now showing a disjunctive pattern.

## ﻿Introduction

The *Pardosaatrata* species-group is one of the smallest ones in the most specious genus of Lycosidae. Currently, three species are considered in this group (Dondale and Redner 1987) vs. 528 in the whole genus (WSC 2023). This taxon was first recognized by Zyuzin (1979). He considered it as a subgroup of the *lapponica* group, although he noted that *P.atrata* is the most deviating species: “other species are mountainous while *P.atrata* is sphagnum dweller” (Zyuzin 1979), and in his work on the Palaearctic *Pardosa*, Zyuzin placed two species in the *atrata* subgroup: *P.atrata* Thorell, 1873 and *P.narymica* Savelyeva, 1972. Dondale and Redner (1987), in their revision of the Nearctic *Pardosa*, added an additional species in this group, *P.fuscula* (Thorell, 1875). *Pardosaatrata* and *P.fuscula* have wide ranges in the Palaearctic and in the Nearctic, respectively (WSC 2023), while *P.narymica* is known only from the type locality in northeastern Kazakhstan (Mikhailov 2021). These three species have never been treated in the same work and have never been compared side by side. It is worth mentioning that the embolic division has never been illustrated for *P.atrata* and *P.narymica*.

I collected *P.atrata* in many localities from Finland to Chukotka Peninsula and south to Mongolia, and *P.fuscula* in several localities in the Yukon Territory and Washington State. All specimens were caught/collected on bogs, especially with *Sphagnum*, or in wet places near water bodies. While collecting spiders near Lake Sevan (Armenia) in a small boggy place, I caught two specimens of *Pardosa* similar in general appearance and habitat preference to *P.atrata*. Study of these specimens revealed that they belong to a new species. Lake Sevan is located far from the known range of the *atrata* group-over 1700 km by aerial distance and about 15° south of the nearest locality in the Europe (Fig. 6). The goal of this paper is to describe a new species, to compare it with other species of the *atrata* group, and to evaluate the distribution of *Pardosaatrata*.

## ﻿Material and methods

Specimens were photographed using a Canon EOS 7D camera attached to an Olympus SZX16 stereomicroscope, and a JEOL JSM-5200 scanning electron microscope at the Zoological Museum of the University of Turku. Digital images were stacked using CombineZP and edited using CorelDraw graphic design software. Figures of the species were made at different times, resulting in variations in their styles. Lengths of leg segments were measured on the dorsal side. All measurements are given in mm. The distribution map is based on the literature and personal data. Not all localities in Fennoscandia and the Nearctic are shown. The type material will be deposited in the Zoological Museum of Moscow State University (**ZMMU**).

### ﻿Abbreviations

**IBPN** Institute for Biological Problems of the North, Magadan, Russia;

**ZMUT**Zoological Museum, University of Turku, Finland.

## ﻿Species survey

### ﻿ Pardosa armeniaca sp. nov.

Taxon classificationAnimaliaAraneaeLycosidae

284535BB-38E3-5B39-9C7B-E68749A70659

https://zoobank.org/B1671366-CB62-4998-9922-C5053BDEAC5C

**Figure 1.**
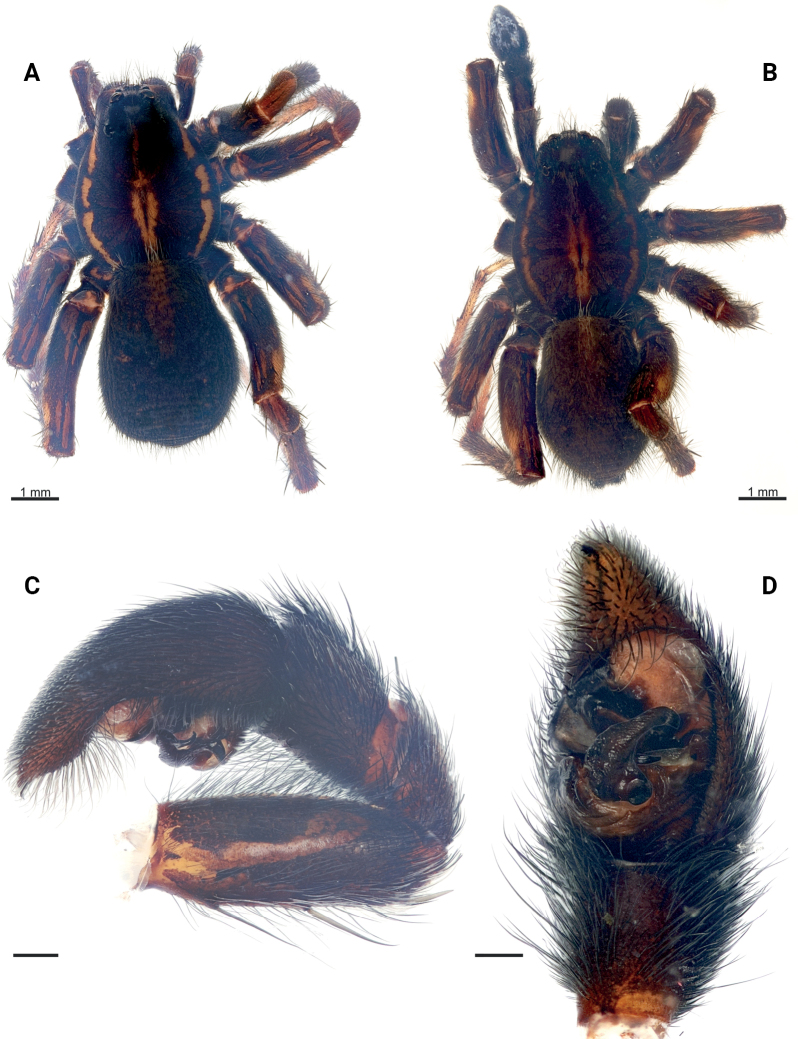
*Pardosaarmeniaca* sp. nov. **A** paratype female **B–D** holotype male **A, B** habitus, dorsal **C** whole palp, retrolateral **D** terminal part of palp, ventral. Scale bars: 0.2 mm, if not otherwise indicated.

**Figure 2.**
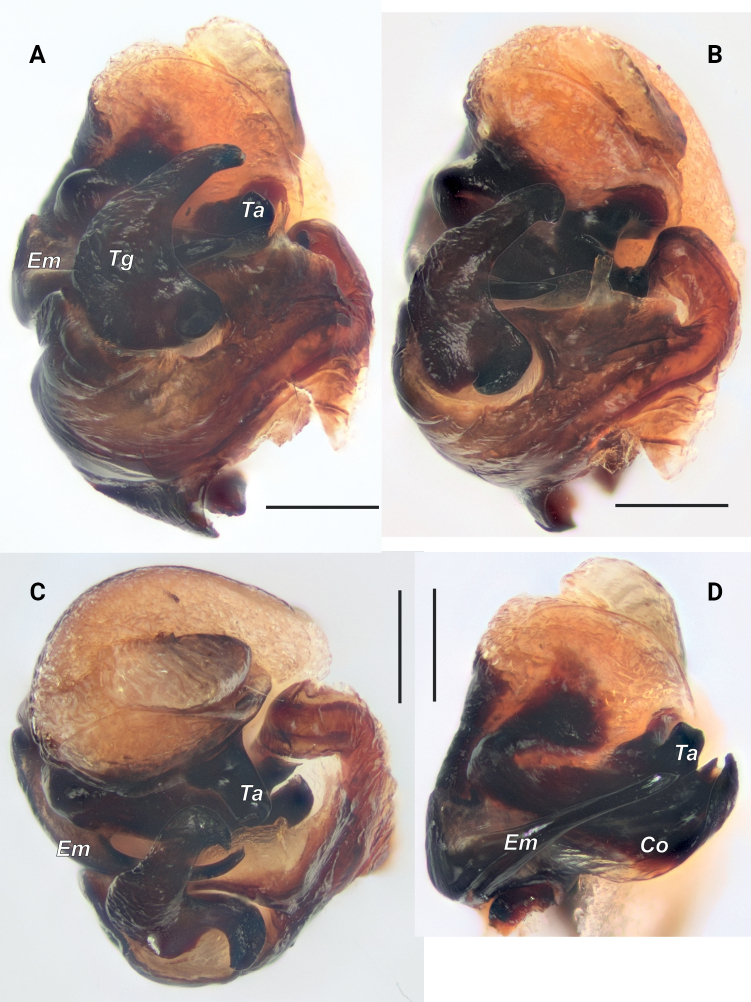
Bulb of *Pardosaarmeniaca* sp. nov. **A** ventral **B** retro-ventral **C** anterior **D** embolic division, ventral. Abbreviations: *Co* – conductor, *Em* – embolus, *Ta* – terminal apophysis, *Tg* – tegular apophysis. Scale bars: 0.2 mm.

**Figure 3.**
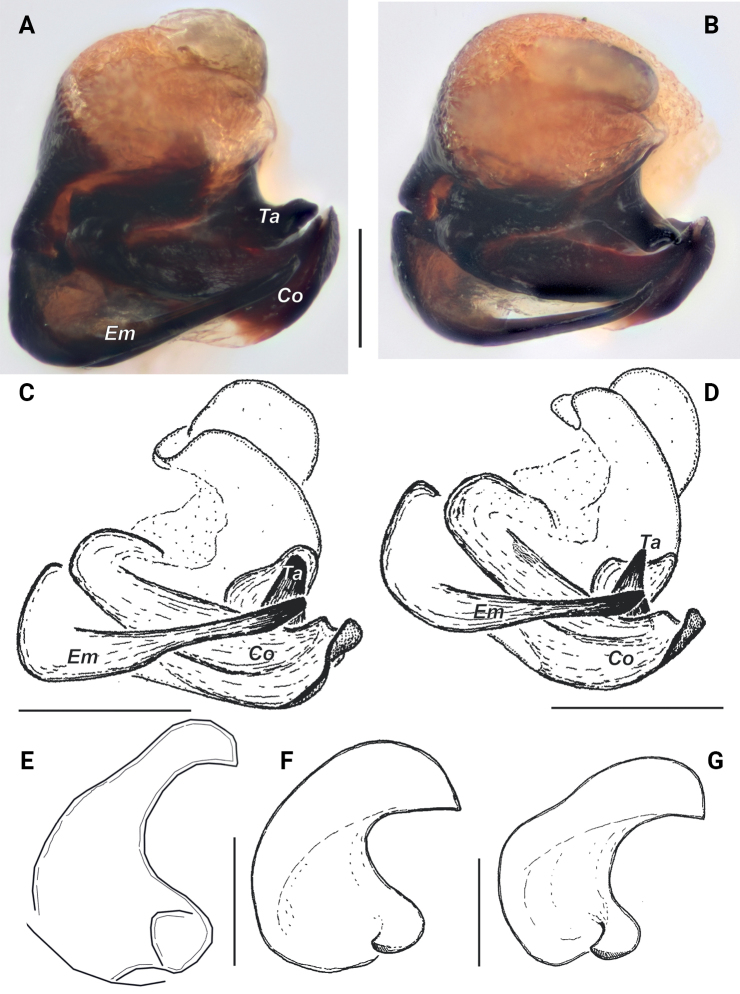
Embolic division (**A–D**) and tegular apophysis (**E–G**) of *Pardosaarmeniaca* sp. nov. (**A, B, E**), *P.atrata* (**C, F** from Polar Ural) and *P.fuscula* (**D, G** from Washington State). **A, C–G** ventral **B** anterior. Abbreviations: *Co* – conductor, *Em* – embolus, *Te* – terminal apophysis. Scale bars: 0.2 mm.

Figs 1 , 2 , 3A, B, E , 5A–C , 6

#### Types.

***Holotype*** ♂ and ***paratype*** ♀ (ZMMU), Armenia, Gegharkunik Prov., Lake Sevan, env. of Tsovagyugh, Dzknaget River mouth, 40°36'53.4"N, 44°58'10.8"E, 1920 m, bog, 8.05.2021 (Y.M. Marusik).

#### Etymology.

The species name is derived from the terra typica.

#### Diagnosis.

**Figure 4.**
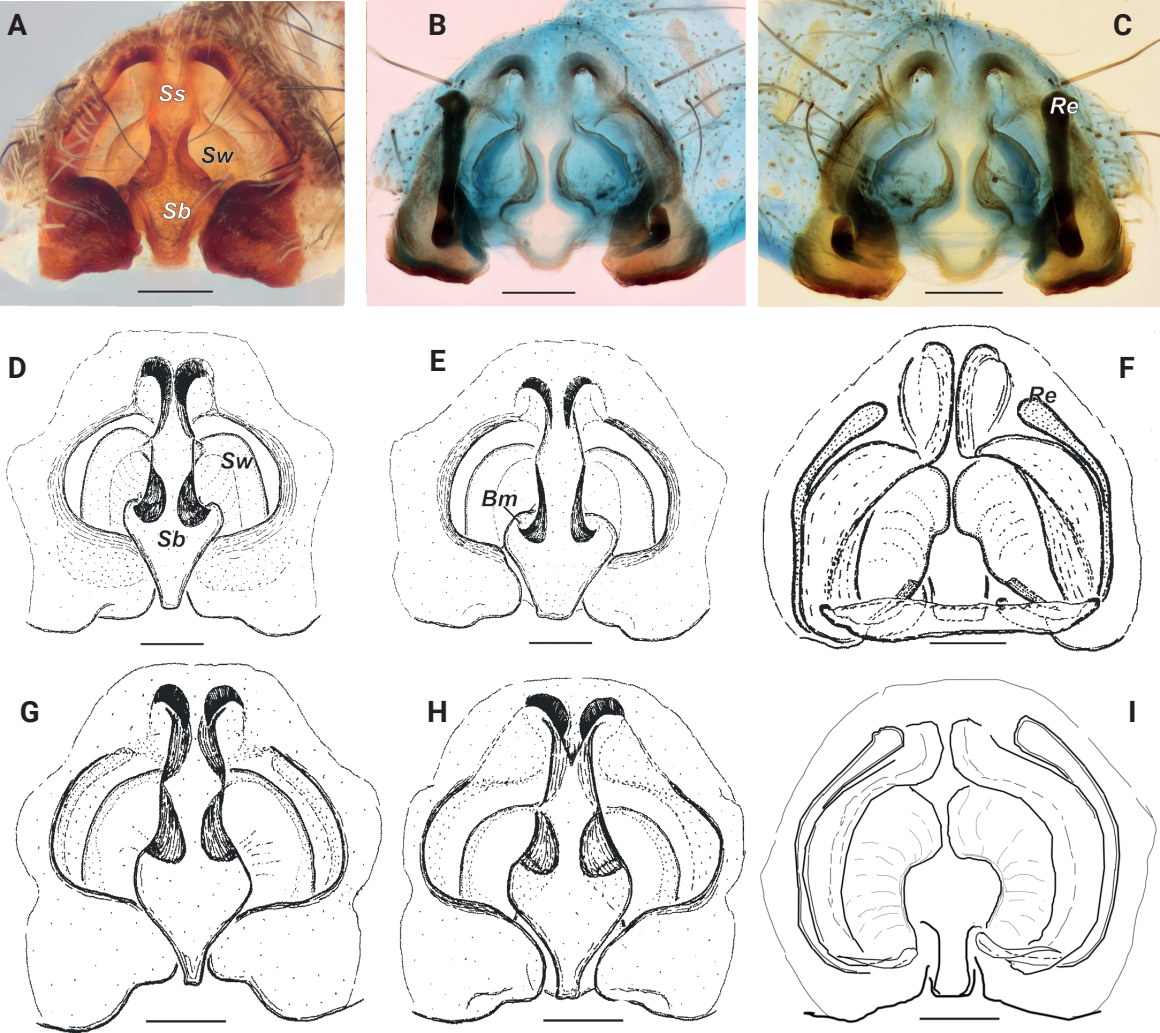
Epigyne of *Pardosaarmeniaca* sp. nov., *P.atrata*, and *P.fuscula***A–C***P.armeniaca* sp. nov. **D–F***P.atrata***G–I***P.fuscula***A, B, D, E, G, H** ventral **C, F, I** dorsal **D** from Utsjoki, Finland **E** from Yamalo Nenets Okrug, Russia **G, H** from Washington State. Abbreviations: *Bm* – rounded margin of septal base, *Re* – receptacle, Sb – septal base, *Ss* – septal stem, *Sw* – septal wing. Scale bars: 0.2 mm.

**Figure 5.**
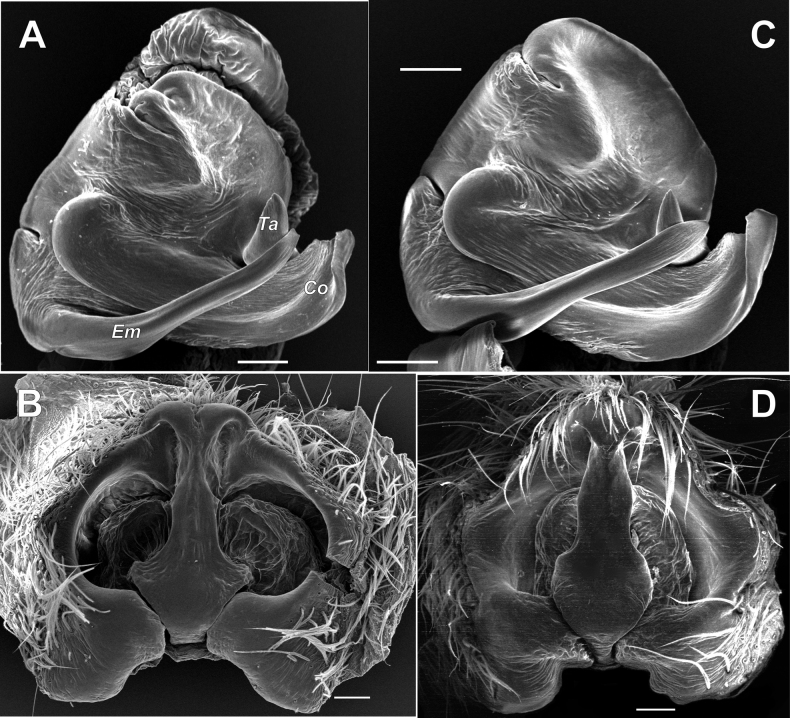
Embolic division and epigyne in *Pardosaatrata* and *P.fuscula***A, B***P.atrata* from Oulu **C, D***P.fuscula* from Washington State **A, C** embolic division, ventral **B, D** epigyne, ventral. Abbreviations: *Co* – conductor, *Em* – embolus, *Te* – terminal apophysis. Scale bars: 0.1 mm.

The male of the new species differs from those of other species of the *atrata*-group in having longer anterior arm of the tegular apophysis (cf. Fig. 3E, F, G), relatively longer and more abrupt terminal apophysis directed antero retrolaterally (vs directed almost anteriorly) and different shape of the conductor (cf. Figs 2C, D, 3A, B). The female of *P.armeniaca* sp. nov. is very similar to that of *P.atrata* but differs in having relatively short, straight, and almost parallel receptacles (vs longer, converging, and roundly bent; cf. Figs 4F, I, 5B, C).

**Male.** Total length 7.5. Carapace 4.1 long, 2.85 wide. Dark coloured. Carapace dark brown, with thin, light-brown median stripe divided by dark median line, with partly broken submarginal light stripe thinner than dark marginal stripe; cephalic light spot absent. Sternum black. Abdomen almost uniformly dark brown. Legs lacking annulation, coxae–tibiae dark brown, metatarsi and tarsi brown.

Leg lengths:

**Table:** 

	Fe	Pt	Ti	Mt	Ta	Total
Palp	1.5	0.7	0.66	—	1.36	4.22
I	2.5	1.26	2.0	2.1	1. 5	7.86
II	2.46	1.2	1.9	2.1	1. 5	7.66
III	2. 5	1.6	1.86	2.4	1.5	7.36
IV	3.06	1.3	2.56	3.7	2.0	12.62

Palp as in Figs 1C, D, 2, 3A, B, E; dark brown with light retrolateral stripe on femur; femur 2.7 times longer than wide; patella 1.3 times longer than wide, tibia almost as wide as long; cymbium as long as femur (in lateral view), tip with 1 claw; bulb 1.3 times longer than wide, tegular apophysis (*Tg*) large, with long anterior arm, ca. as long as palea; terminal apophysis (*Ta*) claw-like (in lateral view) and stump-like in anterior view; conductor (*Co*) massive with rounded notch on tip; embolus (*Em*) straight in ventral view, roundly bent in anterior; tip not modified.

**Female.** Total length 8.0. Carapace 4.1 long, 3.15 wide. Colouration as in male, with wider submarginal stripes as wide as marginal stripes. Legs and leg segment length as shown in table below. Femur I with 2 prolateral spines, femur II with 1; tibiae I–IV with 3 pairs of ventral spines including apical; metatarsus I with 3 pairs of ventral spines including apical.

Leg lengths:

**Table:** 

	**Fe**	**Pt**	**Ti**	**Mt**	**Ta**	**Total**
I	2.5	1.26	1.96	2.0	1.36	9.08
II	2.5	1.26	1.8	2.0	1.36	8.92
III	2.5	1.2	1.86	2.26	1.4	9.22
IV	3.26	1.3	2.5	3.76	1.9	12.72

Epigyne as in Fig. 5A–C; epigynal plate ca. 1.3 times wider than long; fovea trapezoidal; ca. 2 times wider than long; septum as long as fovea wide, rounded margin of septal base absent; septal wings (*Sw*) small, ca. halflength of septal stem (*Ss*). Receptacles (*Re*) long (>10 times longer than wide) and straight, almost parallel to axis of epigyne, anterior part slightly widened.

### ﻿ Pardosa atrata

Taxon classificationAnimaliaAraneaeLycosidae

Thorell, 1873

21457E8E-FE4E-5A32-8D19-0579B3C23302

**Figure 6.**
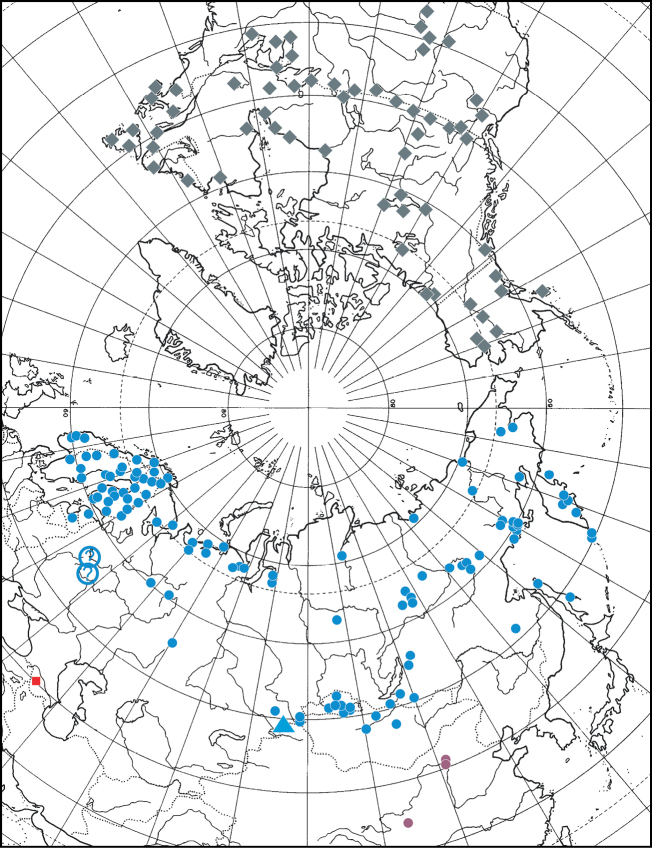
Distribution map with records of *Pardosaarmeniaca* sp. nov. (red square), *P.atrata* (blue dot), *P.fuscula* (grey diamond), the type locality of *P.narymica* (blue triangle), doubtful records of *P.atrata* (question mark), and Chinese records of *P.atrata* that may belong to other species (pink dots).

Figs 3C, F , 4A, B , 5D–F , 6

Lycosa lapponica Thorell, 1872: 273 (♀, not ♂).
Lycosa atrata Thorell, 1873: 576 (♂♀).
Lycosa camtschadalica Kulczyński, 1885: 52, pl. 11 fig. 31 (♂♀).
Lycosa atrata : Kulczyński 1926: 36 (synonymized L.camtschadalica).
Lycosa atrata : Holm 1947: 35, fig. 3b, pl. 6 figs 70, 71, pl. 10 fig. 44 (♂♀).
Pardosa narymica Savelyeva, 1972: 460, fig. 1д, б–в (♂♀), syn. nov.
Pardosa atrata : Almquist 2005: 211, fig. 210a–f (♂♀).
For complete list of taxonomic references see WSC (2023).

#### Material examined.

Norway: 10♂♀ (ZMUT), Finnmark, Varangerbotn, palsa bog, 19.06–20.09.1972 (S. Koponen). Finland: 1♂ (ZMUT), Utsjoki, Karigasniemi, ca. 69°24'N, 25°51'E, 1–15.07.2004 (S. Koponen); 23♂♀ (ZMUT), Pöytyä, Kontolanrahka, 60°43'N, 22°36'E, 22.05–12.09.1976 (S. Koponen); 10♂ 5♀ (ZMUT), Oulu area, Ruukki, Revonneva bog, 60°40'N, 25°05'E, 30.05–30.9.1977 (S. Koponen). Russia: 6♂ 4♀ (IBPN), Arkhanglesk Oblast, Dolgiy Island, 69°N, 59°E, 3–28.06.2004 (O.L. Makarova); 45♂♀ (IBPN), Nenets Okrug, Pakhancheskaya Guba, Matyul-Salya Cape, 68°31'N, 57°19.5'E, 25.07–5.08.2015 (O.L. Makarova, M. Bizin). 1♂ 3♀ (ZMUT), Yamalo-Nenets Okrug, Oktyabrsky, 66°41'N, 66°34'E, sandy shore, 12–13.07.1994 (S. Koponen); 13♂ 5♀ (IBPN), Yamalo-Nenents Okrug, 73 km NE of Labytnangi, foothils of Kharcheruz’ Mt Range, Longotiegan River, ~175 m, 67.3°N, 66.72°E, 1–30.07.2015 (V.K. Zinchenko); 98♂♀ (IBPN), Yamalo-Nenents Okrug, Tazovski Vil., 67°27'N, 78°42'E 27.06–21.07.2008 (M.A. Khrisanova). 1♂ (IBPN), Krasnoyarsk Prov., lower flow of Kotui R., 71°24'N, 103°E, 20.06–5.07.2010 (O.A. Khrulyova). 1♂ (IBPN), Sakhalin, Okha Dist, Pil’tun Bay, 53°00'N, 143°11'E, 06–07.1991 (A.M.Basarukin). 5♂ 7♀ (IBPN), Yakutia, Kolyma River mouth, ca 69°N, July 1999 (A.V. Alfimov). Only some material studied is listed.

#### Note.

In the entry for *P.atrata*, WSC (2023) refers to Schenkel’s (1951: 25 [♀]) record of *P.lapponica* (Thorell, 1872) from Banff National Park (Alberta, Canada) as belonging to *P.atrata*. Schenkel (1951) compared his specimens (females) with those from the eastern Alps (Tyrol). In fact, *P.lapponica* is absent in the Alps, but there exists the closely related species, *P.cincta* (Kulczyński, 1887), which was earlier considered as a junior synonym of *P.lapponica*. On the other hand, *P.lapponica* in the Nearctic is restricted to the north (north of 58°N; Dondale and Redner 1986), and *P.concinna* (Thorell, 1877), the only similar species with an almost identical epigyne, is known in southern Canada and even reaches the highlands in New Mexico (Dondale and Redner 1986). Therefore, Schenkel’s (1951) record of *P.lapponica* most likely refers to *P.concinna* and not to *P.atrata*, which is unknown from the Nearctic or the European Alps.

#### Diagnosis.

This species differs from other *atrata*-group species in having the shortest tegular apophysis (cf. Fig. 3E–G) and receptacles of intermediate length (Fig. 5A–I). The epigyne of *P.atrata* differs from that of *P.armeniaca* sp. nov. in having the receptacles roundly bent (vs straight), and *P.atrata* can be distinguished from the Nearctic *P.fuscula* by the relatively shorter receptacles (cf. Fig. 5F and I), as well as by the base of septum, which has well-developed, rounded margins (*Bm*) (vs stem gradually turns to the base).

#### Description.

See Holm (1947) and Almquist (2005).

#### Comments.

Two names *narymica* and *P.atrata* are synonymised here based on the similarity of the epigynes. *Pardosanarymica* was described based on the holotype female and male paratype from East Kazakhstan Oblast. The types of this species were lost (destroyed) during shipment (Ovtsharenko pers. comm.). Judging from the original figures, the male was mismatched with the female; the male it has tegular apophysis like in *P.lapponica* (Thorell, 1872) and was an undescribed, related species occurring in the region (personal data). In addition, *P.atrata* is known in the adjacent Altai (Azarkina and Trilikauskas 2013). On the other hand, *P.camtschadalica* synonymized by Kulczyński (1926) without comparison with *P.atrata* may be a valid species.

#### Type locality.

The types are from Sweden, Härjedalen Province (ca 62.26°N, 13.5°E, several syntypes) and one female is from Finnish Lapland (ca 68.448°N, 22.484°E) (Thorell 1873).

#### Distribution.

*Pardosaatrata* has a trans-Palaearctic distribution and while restricted to the north in Europe, it occurs rather far south in Asia, reaching Inner Mongolia (Fig. 6). The southernmost localities in Europe lie at about 54°N (Fig. 6). The record from Moscow Oblast (Sytshevskaja 1935) is doubtful and not confirmed by museum specimens or recent records (Mikhailov pers. comm.).

### ﻿ Pardosa fuscula

Taxon classificationAnimaliaAraneaeLycosidae

(Thorell, 1875)

D69E3620-4026-5030-90ED-EACC4A7B1DB2

Figs 3D, G , 4C, D , 5G–I , 6

Lycosa fuscula Thorell, 1875: 501 (♀).
Pardosa fuscula : Dondale and Redner 1987: 2, figs 1, 2, 17–19 (♂♀).
Pardosa fuscula : Dondale and Redner 1990: 171, figs 219–223 (♂♀).
Pardosa fuscula : Paquin and Dupérré 2003: 162, figs 1799–1802 (♂♀).
For complete list of taxonomic references, see WSC (2023).

#### Material examined.

Canada, Yukon Territory: 1♀ (IBPN), Kluane Lake, Cultus Bay, 61°11'N, 138°20'W, Rat Lake, pebbly NW bank, 23.07.1993 (Y.M. Marusik); 3♀ (IBPN), Kluane Lake, Christmas Bay, 61°03'N, 138°21'W, 22.07.1993(Y.M. Marusik); 1♀ (IBPN), environs of Carmacks, 62°11'N, 136°22'W, around small lake, willow–*Carex* vegetation, 17.03.1993 (Y.M. Marusik); 1♀ (IBPN), environs of Carmacks, 62°11'N, 136°22'W, oligotrophic bog near small lake, 17.07.1993 (Y.M. Marusik). USA: 9♂ 9♀ (IBPN), Washington State, Chelan Co., Fish Lake, 588 m, 48°N, 121°W, sphagnum bog, 19.05.1996 (Y.M. Marusik).

#### Comments.

Although *P.fuscula* has been treated in eight taxonomic papers, proper figures showing details of the copulatory organs, including the embolic division, were given only by Dondale and Redner (1987, 1990). It is worth mentioning that Banks (1910) considered *P.fuscula* to be a junior synonym of *P.modica* (Blackwall, 1846).

#### Diagnosis.

The male of this species differs from those of the other *atrata*-group species in having a conical terminal apophysis (*Ta*) (vs tip of terminal apophysis rounded; cf. Fig. 3E–G). The female of *P.fuscula* can be distinguished by the lack of round lateral extensions of the septal base (*Bm*) (vs present) and also in having relatively longer receptacles (cf. Fig. 5 C, F, I).

#### Description.

See Dondale and Redner (1987, 1990).

#### Type locality.

Strawberry Harbour (55.149°N, 59.023°W), Labrador, Canada.

#### Distribution.

This species is restricted to the Nearctic (Fig. 6) and occurs from westernmost Alaska to easternmost Canada, from the Mackenzie River mouth (ca 69°N) south to northern New Mexico (ca 36°N).

## Supplementary Material

XML Treatment for Pardosa armeniaca

XML Treatment for Pardosa atrata

XML Treatment for Pardosa fuscula

## ﻿Additional information

### Author ORCIDs

Yuri M. Marusik https://orcid.org/0000-0002-4499-5148

### Data availability

All of the data that support the findings of this study are available in the main text.
